# Breast Cancer and miR-SNPs: The Importance of miR Germ-Line Genetics

**DOI:** 10.3390/ncrna5010027

**Published:** 2019-03-20

**Authors:** Poonam Malhotra, Graham H. Read, Joanne B. Weidhaas

**Affiliations:** Department of Radiation Oncology, David Geffen School of Medicine, University of California, Los Angeles, CA 90001, USA; PMalhotra@mednet.ucla.edu (P.M.); gread@ucla.edu (G.H.R.)

**Keywords:** miR-SNPs, breast cancer, polymorphism, 3′UTR, predictive biomarkers

## Abstract

Recent studies in cancer diagnostics have identified microRNAs (miRNAs) as promising cancer biomarkers. Single nucleotide polymorphisms (SNPs) in miRNA binding sites, seed regions, and coding sequences can help predict breast cancer risk, aggressiveness, response to stimuli, and prognosis. This review also documents significant known miR-SNPs in miRNA biogenesis genes and their effects on gene regulation in breast cancer, taking into account the genetic background and ethnicity of the sampled populations. When applicable, miR-SNPs are evaluated in the context of other patient factors, including mutations, hormonal status, and demographics. Given the power of miR-SNPs to predict patient cancer risk, prognosis, and outcomes, further study of miR-SNPs is warranted to improve efforts towards personalized medicine.

## 1. Introduction

Breast cancer is the second most common form of cancer diagnosed in American women, after skin cancer. It is among the most frequently diagnosed cancers worldwide, with 2.08 million new cases in 2018 (11.6% of all cancer cases) [1]. According to American Cancer Society estimates, an American woman has about a 12% average lifetime risk of developing breast cancer. Worldwide, breast cancer led to about 626,000 mortalities in 2018, representing 6.6% of cancer deaths—the second-leading cause of cancer death among women [1,2]. Etiology of breast cancer varies according to age of onset, menopausal status of women, exposure to hormone replacement therapies (HRTs), and patient ethnicity [3]. These multiple factors have been associated with differences in risk and outcome.

The first documented involvement of microRNAs (miRNAs) in human cancers was when miR-15 and miR-16-1 were found to be downregulated in chronic lymphocytic leukemia (CLL) [4], revealing a new class of oncogenic miRNAs (oncomiRs) differentially regulated in cancer. Since then, numerous other oncomiRs have been identified, including oncogenic [5] and tumor suppressive miRNAs [6]. Atypical miRNA expression varies between tumor types, and can exert a range of functional effects depending on the cellular context [7].

miRNA expression and targeting can be significantly altered by the presence of single nucleotide polymorphisms (SNPs). SNPs are the most frequently encountered point mutations in the genome, with the first genomic SNP being identified as early as 1976 [8]. Since then, over 84 million SNPs have been documented in the human genome [9], including many in miRNA primary sequences, miRNA target sites, or miRNA biogenesis genes, which we refer to as miR-SNPs [10,11]. The overall effect of a miR-SNP on miRNA function depends on its location—miR-SNPs can result in over-expression, degradation, or transcriptional or translational inhibition of miRNAs or targeted mRNA [12].

**Figure 1:**
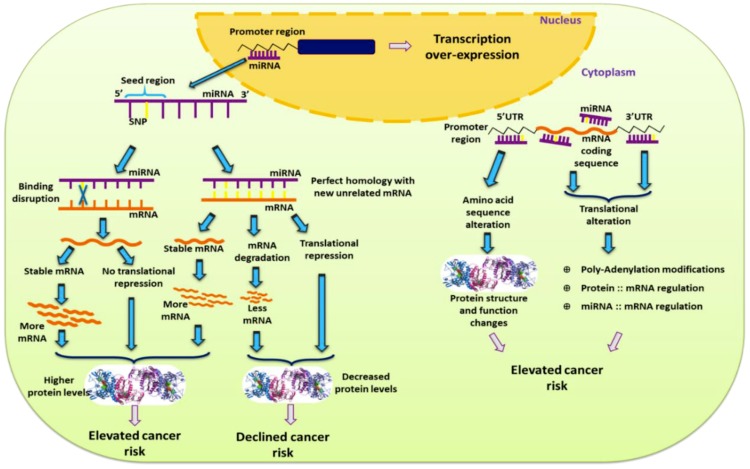
Effect a single nucleotide polymorphism (SNP) exerts in development of cancer risk based on its location in miRNA.

miR-SNPs located in regulatory regions of genes, at miRNA binding sites, or at quantitative trait loci play a major role in gene function and phenotype (Figure 1). Such miR-SNPs can lead to increased risk of multiple types of cancer, including breast cancer [12]. miR-SNPs in seed regions of miRNAs (regions responsible for miRNA-mRNA binding) can disrupt miRNA binding or be lethal. In addition, by altering sequence homology, SNPs can create new interactions between miRNA seed regions and target mRNA [13] (Figure 1). miR-SNPs in the 3′UTRs of mRNA can alter polyadenylation, protein-mRNA interactions, and miRNA-mRNA interactions. These changes can have significant effects on mRNA stability and translation efficiency [14]. Similarly, SNPs in miRNA sequences, including primary and precursor miRNA sequences, can affect production or stability of these miRNAs, as well as miRNA-mRNA interactions, leading to changes in the expression of miRNA target genes [15]. Changes in miRNAs and their mRNA interactions can also be mediated by alterations to miRNA biogenesis genes, which can be brought about by SNPs in proteins responsible for miRNA processing after transcription [16,17]. All of these types of miR-SNP can be prospective candidates as biomarkers for detecting disease susceptibility, determining prognosis for personalized therapeutic regimens, and ensuring the best clinical management of breast cancer patients [7,18,19,20,21]. In this review, we will discuss miR-SNPs found to be important in breast cancer.

## 2. SNPs in miRNA Biogenesis Genes and Breast Cancer

**Figure 2:**
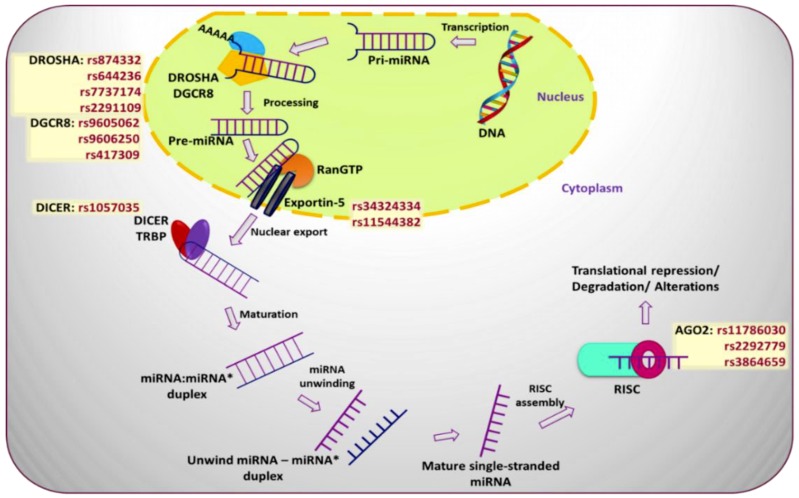
miRNA biogenesis pathway and various single nucleotide polymorphisms (SNPs) occurring at different steps of the pathway that play a pivotal role in prediction of breast cancer risk development.

SNPs affecting proteins responsible for post-transcriptional processing of miRNAs can significantly affect miRNA function. Briefly, miRNAs go through two rounds of enzymatic processing after initial transcription-processes, which implicate miRNA-specific exonucleases, transport proteins, and signaling cascades [22]. The resulting changes to levels of mature miRNA can have significant effects on breast cancer. Significant SNPs have been observed in 3′UTR, exonic, and intronic regions of miRNA processing genes [21], and at various steps of the miRNA biogenesis pathway [23] (Figure 2).

**DROSHA:** The nuclear RNase III, Drosha, is the core nuclease that executes the initiation step of miRNA processing, cleaving primary miRNAs to release hairpin-shaped pre-miRNAs. A case-control study of the SNPs rs644236 (C > T) and rs7737174 (A > G) within the DROSHA gene demonstrated increased risk of developing breast cancer (OR = 1.27; CI 0.94–1.73, and OR = 1.63; CI 1.01–2.64, for rs644236 and rs7737174, respectively) in Korean post-menopausal women [20]. Another report also associated rs2291109 (A > T) in DROSHA with increased breast cancer susceptibility [Odds ratio (OR) = 0.81; 95% Confidence Interval (CI) 0.66–0.99] in Chinese patients [24].

**DGCR8:** DGCR8 is believed to be an essential component of the pri-miRNA processing complex with Drosha. A DGCR8 SNP, rs9606250 (A > T) is strongly linked to poor disease-free survival (HR = 0.21; CI 0.05–0.84) in Korean breast cancer patients [21]. An additional study in a cohort of 878 Chinese breast cancer patients and 900 controls found that rs417309 (A > G), present in the 3′UTR of DGCR8 mRNA is associated with a higher risk of developing breast cancer (OR = 1.50; CI 1.16–1.93) by altering binding ability of miR-106b and miR-579 to the DGCR8 3′UTR [24].

**XPO5:** Alterations to the expression and epigenetic state of Exportin5 (XPO5), responsible for exporting pre-miRNAs from the nucleus, has been associated with risk of breast cancer [25]. The XPO5 missense SNP rs11544382 (A > G) is significantly linked with increased breast cancer risk (OR = 1.59; CI 1.06–2.39) compared to homozygous controls in Caucasian populations. Women in post-menopausal stages with the variant allele genotypes of rs11544382 and rs34324334 are also highly prone to developing breast cancer due to altered nucleocytoplasmic transport activity (OR = 1.82 and 1.76, respectively). Increased levels of XPO5 promoter methylation correlate with a reduced risk of breast cancer, consistent with tissue array data showing higher expression of XPO5 in breast cancer cells relative to tumor-adjacent and healthy tissue [25]. This demonstrates that genetic and epigenetic changes to the miRNA biogenesis pathway members can have a significant effect on the risk of breast cancer development.

**DICER1:** DICER1 is another RNase III protein located in the cytoplasm that cleaves pre-microRNAs into miRNAs. The SNP rs1057035 (C > T), located in the 3′UTR of DICER1, plays a significant role in disease-free survival (DFS) (HR = 1.72; CI 0.99–2.99) and overall survival (OS) (HR = 2.08; CI 1.01–4.28) in Korean breast cancer patients [21]. This miR-SNPs is hypothesized to be present in the binding site of miR-574-3p, affecting miRNA binding and DICER mRNA expression levels [26]. Notably, the SNP is also observed to be linked with significantly elevated risk of breast cancer progression (1.72-fold change) and a 2.08-fold higher breast cancer-associated mortality [21]. However, the C allele of rs1057035 had been shown to have disparate effects on breast cancer risk, presenting a decreased risk of breast cancer in Asian populations, but not in Caucasian populations (C vs. T: OR = 0.88, CI 0.81–0.95, *p* = 0.002; TC vs. TT: OR = 0.85, CI 0.77–0.93, *p* = 0.001; CC/TC vs. TT: OR = 0.86, CI 0.78–0.94, *p* = 0.001) [27], indicating significant differences in the impact of miR-SNPs based on patient demographics.

**AGO2:** AGO2 is a critical component of the RISC complex that brings miRNAs to their target 3′UTR binding sites. A study was undertaken to assess the effect of 41 SNPs in AGO2 on DFS and OS in 488 Korean breast cancer cases [20]. It was found that rs11786030 (A > G) and rs2292779 (C > G), located in AGO2, resulting in decreased DFS (HR = 2.62; CI 1.41–4.88 and HR = 1.42; CI 1.06–1.92 for rs11786030 and rs2292779 respectively) as well as poor OS (HR = 2.41; CI 1.05–5.50 and HR = 2.94; CI 1.52–5.69 for rs11786030 and rs2292779 respectively) in breast cancer patients [21]. Conversely, SNP rs3864659 (A > C) in AGO2 protected against breast cancer risk (OR = 0.67; CI 0.46–0.96) in a Korean population, as observed by a case-control study involving 559 breast cancer cases vs. 567 controls [20].

## 3. 3’UTR SNPs and Breast Cancer

Numerous SNPs in miRNA binding sites interfere with miRNA target recognition ability, causing dysregulation of target genes by altering miRNA and mRNA interactions [12,14]. When such miR-SNPs occur in an oncogene or a tumor regulatory gene, the resulting alterations in gene regulation can shift cellular homeostasis towards tumorigenesis [12]. Sequence variations in the 3′UTR of genes important in the stress response or DNA repair alter the function of effector proteins, which causes modifications in the ability to repair damaged DNA, increasing breast cancer risk [28]. The following miR-SNP examples delineate the roles of SNPs in miRNA binding sites in the 3′UTRs of important genes and their implications in breast cancer.

**RAD52:** The C allele of SNP rs7963551, positioned in the 3′UTR *let-7* binding site of RAD52, reduces risk of breast cancer (OR = 0.84; CI: 0.75–0.95) [29]. Similarly, Cao and colleagues found that patients with the AC variant allele and CC allele genotypes of rs7963551 had a significantly lower risk of breast cancer (OR 0.684; CI 0.492–0.951 for AC genotype; OR 0.317, CI 0.200–0.503 for CC genotype) (Table 1). Via qRT-PCR and western blot analysis, they also demonstrated that *let-7b* plays a pivotal role in downregulating RAD52 expression in MCF-7 and SKBR-3 cells [28]. In addition, women with a higher number of pregnancies and the A allele of rs7963551 were shown to have significantly higher breast cancer risk (OR 2.63, CI 2.03–3.42) [28].

**BRCA1:** A functional SNP, rs8176318, located in the 3′UTR of BRCA1, has been reported to predict breast (including TNBC) and ovarian cancer risk in a population of Irish women [30]. This SNP is also influenced by estrogen exposure, implying that the variant poses an elevated risk of developing cancer at menopause or upon estrogen withdrawal. The variant TG or TT alleles were compared to a wildtype GG allele in the BRCA1 3′UTR using an in vitro luciferase reporter assay and by immunofluorescence in triple negative breast cancer samples. The authors observed decreased BRCA1 gene expression in TNBC cases bearing TT variants in comparison to GG allele cases. The same results were documented in vitro using TT allele breast cancer cell lines. In accordance with decreased BRCA1 expression, TG or TT allele patients demonstrated elevated breast cancer risk relative to nonvariant patients (OR = 1.4; CI 1.1–1.8). Interestingly, variant genotype cases showed four-fold elevated risk of developing Stage IV disease. On the other hand, an elevated risk of breast cancer in G allele variants was observed in African American women (OR = 9.5; CI 1.01–88.80) with specific risk towards developing TNBC (OR = 12.2, CI 1.29–115.21), emphasizing its utility as a strong genetic marker for predicting potential TNBC risk [31].

Additionally, it has been reported that homozygosity for the A alleles in miR-SNPs rs12516 and rs8176318, located in the 3′UTR of BRCA1, has a significant association (*p* = 0.007) with familial breast and ovarian cancer in a Thai cohort [32]. An additional BRCA1 3′UTR SNP, rs3092995, induces an elevated breast cancer risk in African American women, with the G allele predominant in BC patients, as compared to control subjects (Table 1) [33].

**IQGAP1:** IQGAP1 (encoding IQ motif-containing GTPase-activating protein 1) is regulated by miR-124 via a binding site in its 3′UTR. miR-SNP rs1042538 in the core binding region causes a disruption in this target site sequence. It has been hypothesized that the presence of this miR-SNP in the miR-124 binding site could be predictive of breast cancer risk and prognosis. Zheng and colleagues analyzed the frequency of rs1042538 A/T variants in 1,541 breast cancer cases and 1,598 controls for associated breast cancer risk factors. It was observed that the TT genotype is linked to a lower breast cancer risk as compared to the AA genotype in a Chinese population (*p* = 0.049, OR = 0.78; CI 0.61–0.99), implying that the T allele of rs1042538 protects from breast cancer (Table 2). Conversely, the AA genotype of the binding site miR-SNP is linked with increased breast cancer risk due to downregulation of IQGAP1 protein expression [12].

**ITGB4:** Brendle and colleagues documented the involvement of integrins in breast cancer risk and clinical outcome in a Swedish case-control study. They showed that the presence of the A variant of miR-SNP rs743554 in the ITGB4 3′UTR was strongly associated with specific risk of developing ER-negative breast cancers and worse overall survival in comparison to those with the wildtype alleles (OR 2.09; CI 1.19–3.67) (Table 2). It was also shown by in silico analysis that this SNP impairs miR-34a binding to the ITGB4 3′UTR seed region [36].

**ESR1:** rs2747648 is present in the 3′UTR miRNA binding site of ESR1, leading to increased ESR151 expression levels due to a lack of miR-453-mediated repression [37] (Table 2). A case-control study in a German cohort of 1223 breast cancer families and 1495 controls demonstrated that the T allele variant of this miR-SNP was association with elevated breast cancer risk in pre-menopausal women (OR 0.60; CI 0.41–0.89) [37]. Similarly, other researchers noted that pre-menopausal women bearing C allele genotypes demonstrated a lower risk of developing breast cancer and had depleted levels of *ESR1* in clinical studies [38,39].

**TGFB1:** It has been reported that individuals with the AG genotype of SNP rs334348 in the 3′UTR of TGFBR1 are more prone to breast cancer (OR = 2.2; CI 1.29–4.07; *p* = 0.005). The G allele was shown to be targeted by miR-628-5p with higher efficacy than its A allele counterpart in vitro [14]. The same study observed that SNP rs1982073 in TGFB1 could exert its effect on gene expression by altering interaction with miR-187, and CC carriers of rs1982073 exhibited an increased risk of breast cancer (OR = 1.4, CI = 1.1–2.0; *p* = 0.04) [14]. They also noted that miR-628-5p represses TGFBR1, in a manner dependent on rs334348 variant inside the 3′UTR miRNA target sequence. Expression of TGFBR1 was 80% of control levels in cells with the AA genotype versus 50% of control levels in cells with the CC genotype) (Table 2). In addition, rs1982073 TGFB1 variant was associated with lower TGFB1 expression levels at early tumorigenic stages, while elevated TGFB1 levels were observed in advanced metastatic phases, linked to simultaneous miR-187 expression level fluctuations in the presence of T variant genotypes [14].

**TOPBP1:** Topoisomerase IIb binding protein 1 (TOPBP1) is reported to possess miR-SNP rs115160714. Three miRNAs (miR-3138, miR-4302, and miR-1207-5p) are hypothesized to bind TOPBP1 mRNA at its 3′UTR. CT and TT variants of rs115160714 are associated with higher breast cancer risk in comparison to CC genotype in Caucasian populations (OR 3.54, CI 1.56–8.39; OR 5.40, CI 0.63–46.64, respectively) (Table 2). It was noted that a grade three tumor was more often reported in individual cases bearing a T variant allele. In addition, patients with CT or TT genotypes were observed to have elevated levels of TOPBP1 mRNA and protein [40,41].

**MMP9:** Matrix Metalloproteinases are frequently overexpressed in multiple cancers, and MMP overexpression correlates with increased invasion [42] and poorer prognosis [43]. The C allele of the miR-SNP rs1056628 has been documented to affect miR-491-5p binding to the MMP9 3′UTR, preventing miRNA-mediated inhibition. In vitro analysis demonstrated that cells transfected with C allele-bearing reporter plasmids of the MMP9 3′UTR had significantly lower miR-491-5p binding relative to cells transfected with the wildtype A allele. The same study also demonstrated that the presence of the C allele imparts a significantly higher risk of breast cancer in an Iranian population (OR 3.23, CI 1.34–7.79). Similarly, C allele patients were more likely to have metastases (OR 1.9, CI 1.21–6.8), consistent with higher expressions of MMP9 [44].

## 4. The *KRAS-*Variant and Breast Cancer Risk

Kirsten Rat Sarcoma Viral Oncogene (*KRAS*) is a frequently mutated oncogene and a key member of the MEK signaling pathway. The 3′UTR of *KRAS* contains a *let-7* binding site SNP, rs61764370, referred to in the literature as *KRAS-*variant. Several case control studies have linked the *KRAS-*variant to increased risk of breast cancer, though the effect appears to be dependent on patient context (Table 3). Premenopausal, but not postmenopausal patients with variant T/G or G/G genotypes were shown to have greater risk of TNBC (OR 1.64; CI 0.79–3.43, and OR 0.77; CI 0.51–1.16, respectively) [45]. Further analysis of *KRAS*-variant tumors in this study found an increase in MAPK signaling and a lower expression of *let-7* via NF-κB-mediated activation of LIN-28, and a distinct gene expression pattern for *KRAS*-variant tumors [46]. Notably, the *KRAS-*variant also associated strongly with ER/PR negative premenopausal patients, implying that age and hormonal status play a role in *KRAS*-variant-associated risk [45].

A study in Iranian patients also found that patients with T > G variant genotypes had a higher risk of developing breast cancer relative to TT genotypes (TT vs. GG + GT: OR = 3.55, CI 1.31–9.65, *p* = 0.012). Also, during G-T allele comparison, the G allele was found to be a risk factor for breast cancer (OR = 3.44, CI 1.28–9.29, *p* = 0.013) (Table 3) [47]. Notably, this study did not find significant associations between the *KRAS*-variant and patient age, ER, or HER2 status.

*KRAS-*variant-associated breast cancer risk appears to be impacted by estrogen withdrawal. McVeigh and colleagues observed that withdrawal from hormone replacement therapy (HRT) was linked to a higher rate of TNBC in *KRAS*-variant patients (*p* < 0.001). Furthermore, patients who previously used HRT and had the *KRAS-*variant tended to be diagnosed with higher grade tumors compared to non-variant patients who had previously been on HRT (2.33 vs. 1.98, *p* = 0.029), indicating that overall the *KRAS*-variant is predictive of an aggressive tumor biology in patients with low estrogen or experiencing abrupt estrogen withdrawal (Table 3). They also evaluated the effect of estrogen deficiency on cell transformation ability in vitro using isogenic MCF10A breast epithelial cells bearing *KRAS*-variant and wild type genotypes. The in vitro study results agreed with clinical conclusions that estrogen withdrawal in *KRAS*-variant cells caused oncogenic transformations in isogenic cell lines. *KRAS*-variant TNBC patients also exhibited significantly elevated aromatase and ERβ expression, which are chiefly regulated by *let-7* that is lower in *KRAS*-variant tumors [48].

Similarly, Cerne and colleagues reported the association of the *KRAS*-variant with HER2 overexpression in breast cancer in postmenopausal women. While prevalence of the *KRAS*-variant was similar in hormone receptor (HR) + and HR-cases, 3 times as many variant patients had HER2 + (42.9%) versus HER2-(13.3%) tumors, and *KRAS*-variant tumors had significantly higher histopathological grades [49]. In contrast, a separate group found 20% of *KRAS-* variant T > G breast cancer cases in a Czech population to be HER2 negative, and 3% of *KRAS-*variant cases to be HER2 positive. However, this study did not segregate data based on patient age or menopausal status. This may imply that *KRAS-*variant status affects HER2 expression in developing breast cancer, which is age dependent [50].

Notably, GWAS studies of a SNP linked to rs61764370 did not find significant association with risk of breast or ovarian cancer [51]. However, well-constructed case control studies that stratify patients by age [52], hormone levels [48], or Her2 status [49,50] have found significant associations between the *KRAS*-variant and BC risk and biology. Together, these data highlight the importance of proper contextualization of miR-SNPs like the *KRAS*-variant when determining patient risk, and suggest that large cohorts with less robust clinical annotation may not be the best place to define the clinical biology and utility of miR-SNPs, which are clearly context dependent, being influenced by environmental conditions, such as estrogen.

## 5. miRNA-Coding SNPs

SNPs within the mature or primary miRNA sequence can have significant effects on miRNA binding or processing [53]. Several SNPs acting directly on miRNA primary sequences or on upstream regulatory elements have been reported with significant effects on risk and prognosis of breast cancer. These miR-SNPs also have different effects between studies, again highlighting the role of patient context in using these SNPs to predict patient risk or prognosis.

**miR27a:** The G allele of miR-SNP rs895819 (A > G), identified on pre-miR-27a, is reported to be associated with a significantly reduced risk of breast cancer in variant patients (OR = 0.88; 95% CI: 0.78–0.99) compared to controls in a German study cohort [54]. Meta-analysis later confirmed that the presence of a G allele was significantly associated with diminished breast cancer risk (G vs. A; OR = 0.91, 95% CI = 0.86–0.97) (Table 3) [55]. However, this analysis found that rs895819 is linked with reduced risk of breast cancer development in Caucasian, but not Asian populations [55]. On the contrary, Feng and colleagues observed that rs895819 SNP was linked to an elevated risk of breast cancer in Asian populations (AG + GG vs. AA: OR = 1.24; CI 1.03–1.50, *p* = 0.02) [56]. The G variant of this SNP has been shown to alter the secondary structure of pre-miR-27a, resulting in reduction of familial breast cancer risk in younger women (age < 50 years) [57]. It has been reported that the presence of a G allele variant at a terminal position in the pre-miR-27a hairpin could affect secondary structure, leading to cleavage inhibition and impaired processing. This results in the reduced expression of mature miR-27a, exerting a protective effect against breast cancer development, as observed in a Chinese case-control study (OR = 0.535; CI 0.321–0.891) (Table 3) [58].

**miR-196a2:** pre-miR-196a2 has a SNP, rs11614913, whose CC/CT genotypes have been linked to elevated breast cancer susceptibility in Chinese women, as assessed by a case-control study (OR = 2.20; CI = 1.19–4.09, *p* < 0.01) and a clinical pathological study (*p* < 0.01) [59]. A variant C allele SNP causes impairment of mature miRNA expression, leading to decreased mature miR-196a2 levels and increased expression of the miR-196a2 target HOXBP [19,60]. The miR-SNP-induced decline in miRNA expression could be utilized as a predictive biomarker for evaluating breast cancer risk in Chinese populations [59]. However, other studies have implied that rs11614913 polymorphism predicts decreased risk of breast cancer in some populations [61]. Meta-analysis of 16 studies showed decreased risk of breast cancer in Caucasian variant patients (OR = 0.93, CI 0.87–1.00, *p* = 0.044), with no significant effects on risk in overall populations [62]. In contrast, several meta-analyses reported that CC genotype-bearing individuals of this SNP were more prone to breast cancer risk [OR = 0.906, CI = 0.825–0.995] (Table 3) [57,63]. Based on these conflicting results, the effects of miR-196a2 on breast cancer risk may be contextually dependent on other patient factors.

**miR-499:** The pre-miR region of miR-499 encodes a SNP, rs3746444, with alleles A and G, in which variant G induces a higher risk (OR = 1.25; CI 1.02–1.51) of developing breast cancer in Chinese populations but not in Caucasian populations (Table 3) [64]. In addition, the G allele variant of rs3746444 SNP was shown to be associated with a higher breast cancer risk (OR = 1.10; CI 1.01–1.20) in Asian populations, as assessed by meta-analysis [65]. Inversely, Asian populations bearing the T allele of the miR-SNP are predicted to have reduced breast cancer risk, while Caucasian populations are observed to be at an elevated risk of breast cancer for the same variant. This demonstrates the importance of considering ethnicity variations under consideration, while determining breast cancer risk [66].

## 6. Conclusions

Use of miRNA-binding site SNPs as predictive biomarkers holds immense potential as it allows risk stratification for cancer development and cancer biology. This opens up additional avenues of designing efficient prevention strategies, and ensuring cost-effective applications of advanced diagnostic tests [72]. Work remains to be done to get a greater understanding of the function of miR-SNPs in context, since risk appears to be dependent on factors such as patient age, hormone status, and ethnicity. Additionally, the prognostic potential of SNPs in several key miRNA regulatory pathways has yet to be determined. For example, recent research has highlighted the utility of noncoding RNA networks on both miRNA function [73,74] and as breast cancer biomarkers [75,76], but no current studies have linked miR-SNPs in competing endogenous RNAs to breast cancer risk or prognosis. Elaborate and well-characterized case-control studies covering diverse populations of varying ethnicities and from geographic locations, including studies on novel targets, will be an invaluable asset in developing microRNA target SNP-based gene analysis [13]. These studies could distinguish specific contexts for miR-SNPs, whether in 3′UTRs, miRNA biogenesis genes, or in miRNAs themselves, to serve as predictive markers for breast cancer risk and prognosis. An in-depth knowledge of miR-SNP working mechanisms can aid in categorizing individuals according to cancer susceptibility and prediction of subtypes. Further characterization of miR-SNPs and their contextual effects stands to significantly improve patient treatment accuracy and efficacy, as well as aiding in development of predictive biomarkers, both in terms of cancer development and aggressiveness.

## Figures and Tables

**Table 1:** miR-SNPs and DNA Damage Repair Genes Involved in Breast Cancer Risk.

SNP	Affected miRNA	Related Gene	Breast Cancer Association	References
rs7963551 (C allele)	*let-7*	RAD52	Reduced risk	[28,29]
rs8176318 (G > T)	miR-639	BRCA1	Elevated TNBC risk	[30,34]
rs12516	miR-1264	BRCA1	Elevated risk	[32,35]
rs3092995	3′UTR	BRCA1	Elevated risk	[33]

**Table 2:** miR-SNPs and Related Genes Involved in Breast Cancer Risk.

SNP	Affected miRNA	Related Gene	Breast Cancer Association	References
rs743554	miR-34a	ITGB4	Elevated risk	[36]
rs1042538 (A > T)	miR-124	IQGAP1	Reduced risk	[12]
rs2747648	miR-453	ESR1	Elevated risk	[37]
rs334348	miR-628-5p	TGFBR1	Elevated risk	[14]
rs1982073-TGFB1	miR-187	TGFB1	Elevated risk	[14]
rs1056628	miR-491-5p	MMP9	Elevated risk	[44,45]

**Table 3:** miR-SNPs Associated with Breast Cancer Risk Prediction.

SNP	Affected miRNA	Related Gene	Breast Cancer Association	References
rs895819 (A > G)	pre-miRNA-27a	MIR27a	Reduced risk	[54,55,57,58,67]
rs61764370	3′UTR of *let-7*	*KRAS*	Elevated risk	[45,47,48,68,69,70]
rs11614913	pre-miR-196a2	MIR196a2	Elevated risk	[57,59,63]
rs3746444	miR-499	MIR499	Elevated risk	[64,66,71]
